# ﻿A redescription of the poorly known Central American toad *Inciliustacanensis* (Anura, Bufonidae), with a summary of its biology and conservation status

**DOI:** 10.3897/zookeys.1102.79229

**Published:** 2022-05-20

**Authors:** Kathryn McCarthy, Ollie Shinn, Roberto Luna-Reyes, Joseph R. Mendelson III

**Affiliations:** 1 School of Biological Sciences, Georgia Institute of Technology, 950 Atlantic Dr NW, Atlanta, Georgia, 30332, USA Georgia Institute of Technology Atlanta United States of America; 2 Dirección de Áreas Naturales y Vida Silvestre, Secretaría de Medio Ambiente e Historia Natural, Calzada de las Personas Ilustres s/n, Colonia Centro, Tuxtla Gutiérrez, 29000, Chiapas, Mexico Secretaría de Medio Ambiente e Historia Natural Tuxtla Gutiérrez Mexico; 3 Zoo Atlanta, 800 Cherokee Ave SE, Atlanta, Georgia, 30315, USA Secretaría de Medio Ambiente e Historia Natural Atlanta United States of America

**Keywords:** Amphibian, chytridiomycosis, Guatemala, Mexico, Volcán Tacaná, Аnfibio, Guatemala, México, quitridiomicosis, Volcán Tacaná

## Abstract

Based on examination of most of the existing museum specimens of the rare bufonid frog *Inciliustacanensis*, we present a redescription and new diagnosis for this species. The species is limited to small region of the Pacific chain of volcanoes in southeastern Chiapas, Mexico, and adjacent areas of Guatemala. The species has not been observed in the wild since 1984 and may have been reduced or eliminated by regional epidemics of chytridiomycosis.

## Abstract (fr)

Basándonos en la revisión de la mayoría de los especímenes de museo existentes del raro sapo bufónido *Inciliustacanensis*, presentamos una redescripción y una nueva diagnosis para esta especie. La especie se limita a una pequeña región de la cadena de volcanes del Pacífico en el sureste de Chiapas, México, y áreas adyacentes de Guatemala. La especie no se ha observado en la naturaleza desde 1984 y puede haber sido reducida o eliminada por epidemias regionales de quitridiomicosis.

## ﻿Introduction

*Incilius* (= *Bufo*) *tacanensis* was described by Philip Smith in 1952. The original description was based on eight specimens from the vicinity of Volcán Tacaná from both Chiapas, Mexico, and Guatemala. Smith (1952) included a photograph of the preserved holotype (UMMZ 88359) and the paper serves as a complete and accurate description of the holotype and seven paratypes. However, except for listings on regional or national checklists, there have been no reports on any aspect of the biology of the species. In Mexico, *Inciliustacanensis* is not considered at risk in the Norma Oficial Mexicana (SEMARNAT, 2010), and is assigned in the low vulnerability category in the Environmental Vulnerability Score (EVS) proposed by Wilson et al. (2013) and Johnson et al. (2015a), and also used by Johnson et al. (2015b) for the herpetofauna of Chiapas. The species is listed as Endangered on the IUCN Red List (IUCN SSC Amphibian Specialist Group, 2020), based on the criteria of its small geographic ranges (approx. 1313 km^2^) and continuing loss of habitat in the region. The Red List assessment mentions that amphibian chytridiomycosis may represent a conservation threat for the species but owing to the absence of any recent observations or records in collections, the disease has not been formally documented. The Red List assessment identifies research needs for the species as “additional research is needed on its natural history, population size, and distribution.” Using museum specimens, this report aims to address some of these needs.

## ﻿Materials and methods

We recorded traditional morphometric measurements and qualitative descriptions from museum specimens, using the terminology of Mendelson et al. (2012); all measurements presented in mm. We made small incisions in the abdomens of apparently mature individuals to verify sex by direct examination of the gonads and to estimate numbers of eggs in females.

In order to test for the presence of the pathogenic amphibian chytrid fungus *Batrachochytriumdendrobatidis* (Bd), we sampled the skin of preserved specimens with rayon-tipped swabs with plastic handles (Dryswab^TM^ Fine Tip MW113; United States: www.mwe-usa.com). We used a single swab for each specimen, rubbing it five times across each of the following surfaces: ventral surfaces of each hand and foot, pelvic patch, ventrum, lateral and dorsal surfaces of the body. Real-Time PCR assays were conducted by the laboratory of Ana Longo at the University of Florida. To quantify the presence and amount of Bd from each swab sample, we performed quantitative polymerase chain reactions following the protocol of Boyle et al. (2004) using an Applied Biosystems QuantStudio 3 System. We extracted the DNA from swabs using 50 µL of the reagent PrepMan Ultra (Applied Biosystems Cat. 4318930). We used a 146 bp synthetic fragment as a standard for Bd (gBlock, IDTDNA; ITS Hap01; Longo et al. 2013) and created a serial dilution ranging from 10^6^ copies to 10 copies. Swab samples were run in triplicate.

To our knowledge, there are 29 museum specimens of *I.tacanensis* worldwide, discounting mis-identified specimens we encountered in the course of our work. We examined most of these specimens (Appendix 1) either physically or in the form of photographs provided by museum curators. Our morphometric data only includes adult specimens. Museum acronyms follow Sabaj-Perez (2022).

## ﻿Taxonomy

### ﻿ Incilius tacanensis

Taxon classificationAnimalia

P. Smith, 1952

7A665284-E3C7-5188-B4A1-6484858BB057

Figs 1 , 2 , 3

Bufo tacanensis P. Smith, 1952: 176. Holotype: UMMZ 88359. Type-locality: at 1500 m on Volcán de Tacaná, Union Juárez, Chiapas, Mexico.
Cranopsis tacanensis Frost et al., 2006a
Ollotis tacanensis Frost et al., 2006b
Incilius tacanensis Frost et al., 2009

#### Description.

**Figure 1.**
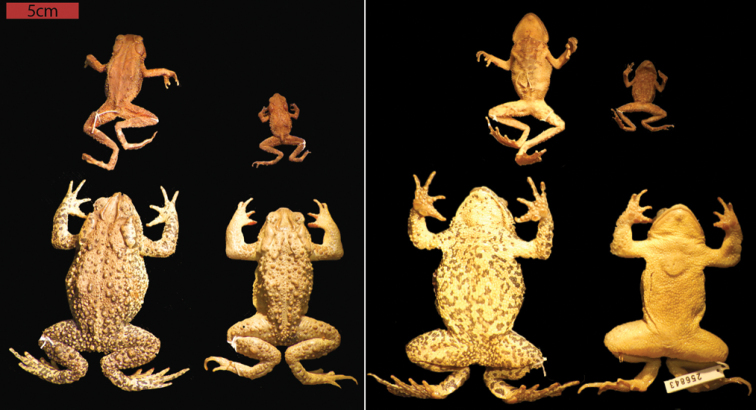
Comparison of dorsal and ventral aspects of typical adult females (left) and males (right) of *Inciliusbocourti* (male: MVZ 256842, female: MVZ 256843 and *I.tacanensis* (male: UIMNH 25473, female: UIMNH 55156). Note the diagnostic differences in size and dorsal skin texture.

Mean SVL in males 36 mm, females 46 mm; cranial crests prominent in most specimens, with the supraorbital and postorbital crests forming an arched L-shaped structure about each eye; preorbital and pretympanic crests present, indistinct; canthal crests present, prominent, extending to above the nostrils; parietal crests prominent, oriented sharply posteromedially, extending to near midline of body; supratympanic crest absent; suborbital crest present but indistinct in some individuals; tympanum is not externally visible; tibia lengths in males range from 43–51% of SVL in males, 40–42% SVL in females; foot length ranges from 43–55% SVL in males, 41–44% in females. Webbing on the foot extends to the tip of every toe, except Toe III, which is webbed only to the second subarticular tubercle. Outer metatarsal tubercle small, rounded, elevated and non-keratinized; inner metatarsal tubercle larger, ovoid, and also non-keratinized. Tips of digits possess small, rounded tips. Morphometric variation is summarized in Table 1, and adult specimens are illustrated in Fig. 1.

**Table 1.** Morphometric variation in adult *Inciliustacanensis.* Mean ± 1 SD above range (in parentheses); all measurements in mm.

Variable	Females *N* = 15	Males *N* = 2
Snout-vent length	51.2 + 4.9 (38.5–57.2)	— (35.3–37.3)
Tibia length	21.8 + 1.4 (18.9–23.6)	— (16.1–18.3)
Foot length	22.0 + 2.1 (17.9–24.8)	— (16.1–19.3)
Head length	16.9 + 1.2 (13.7–17.9)	— (12.1–13.1)
Head width	17.5 + 1.5 (14.1–19.8)	— (12.5–13.3)
Eye diameter	5.8 + 0.7 (4.4–6.9)	— (4.1–4.6)
Eye-nostril distance	4.3 + 0.4 (3.3–4.9)	— (3.7–4.0)
Parotoid length	8.1 + 1.0 (7.1–10.5)	— (5.2–6.0)
Parotoid width	5.2 + 0.6 (3.9–6.3)	— (3.6–3.8)

Texture of the dorsal skin is smooth with scattered small, sharply pointed tubercles, becoming more numerous and dense, laterally and on the limbs. The ventral skin is roughly granular, with weakly pointed tubercles. The lateral row of tubercles is present as a series of small, sharply pointed tubercles that are slightly larger than similar, unorganized proximal tubercles. The parotoid glands are large, rounded, about 1.25 × diameter of eyelid. Tibial and rictal glands are absent. Vocal slit unilateral.

In preservative, dorsal coloration is dull brown with dark brown lateral stripes following the lateral tubercles; some cream spots present on legs in some specimens. Ventrum is dark cream with a diffuse dark brown marbled pattern that extends onto the legs. In general, males tend to be more uniform dull brown than are the moderately patterned females.

#### Diagnosis.

**Figure 2.**
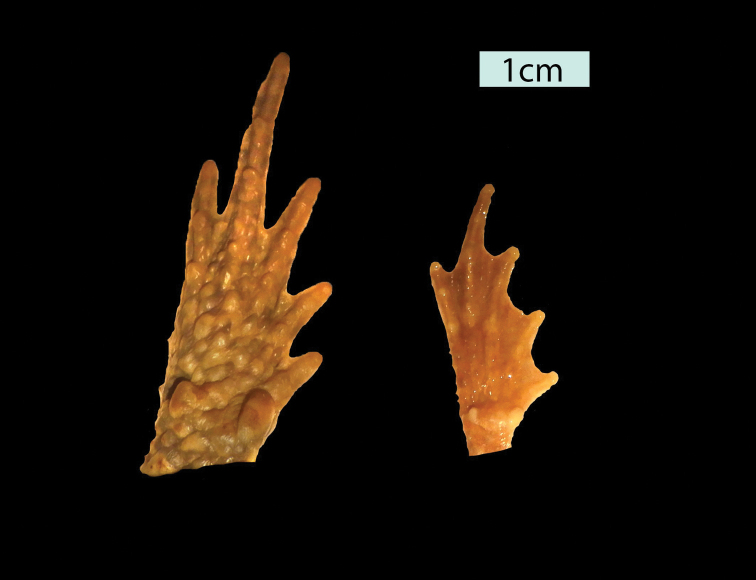
Details of the webbing of the feet of *Inciliusbocourti* (left; MVZ 256842) and *I.tacanensis* (right; CAS 70691). The webbing is more extensive on the feet of *I.tacanensis*.

**Figure 3.**
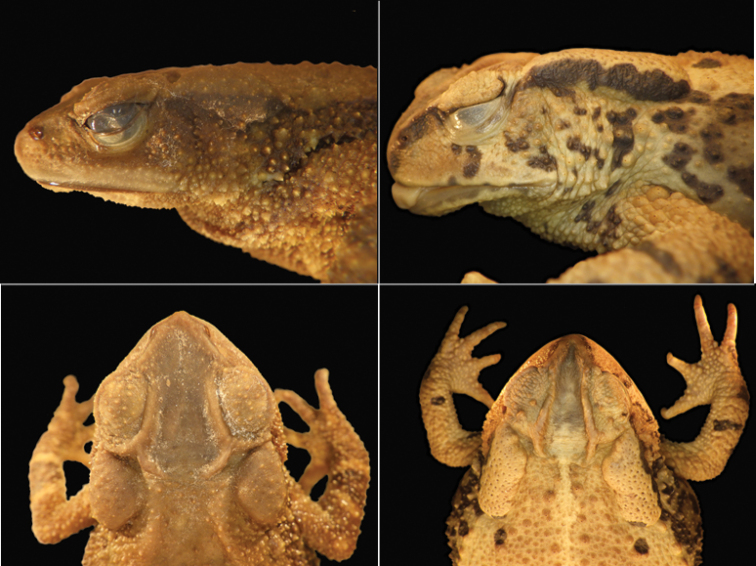
Details of the heads of adult females of *Inciliustacanensis* (left: MVZ 159445) and *I.bocourti* (right: UTA A-13008), showing diagnostic differences in the cranial crests, parotoid glands, and general shape. *Inciliusbocourti* is a much larger species than is *I.tacanensis* (see Fig. 1), so these images are not at the same scale in order to facilitate direct comparisons.

No other bufonid in Mexico or Guatemala has webbing on the feet as extensively developed as in *I.tacanensis* (Fig. 2). Within the range of this species, only *Inciliusbocourti* (Brocchi, 1877) also lacks an externally evident tympanum. *Inciliusbocourti* differs from *I.tacanensis* by lacking vocal slits (vs. present, unilateral), having little webbing on the feet (vs. extensive), reaching sizes up to 70 mm in males and 80 mm in females (vs. 37 mm, 57 mm), by having very large, distinctly oval parotoid glands with length more than 2 × diameter or eyelid (vs. rounded, about 1.25 × eyelid). *Inciliusbocourti* is strongly sexually dimorphic in coloration, with males being nearly uniform greenish yellow and females being dark reddish brown. Based on museum specimens, *I.tacanensis* appears to be generally uniformly dull brown. The heads of the two species are illustrated in Fig. 3.

#### Distribution and ecology.

**Figure 4.**
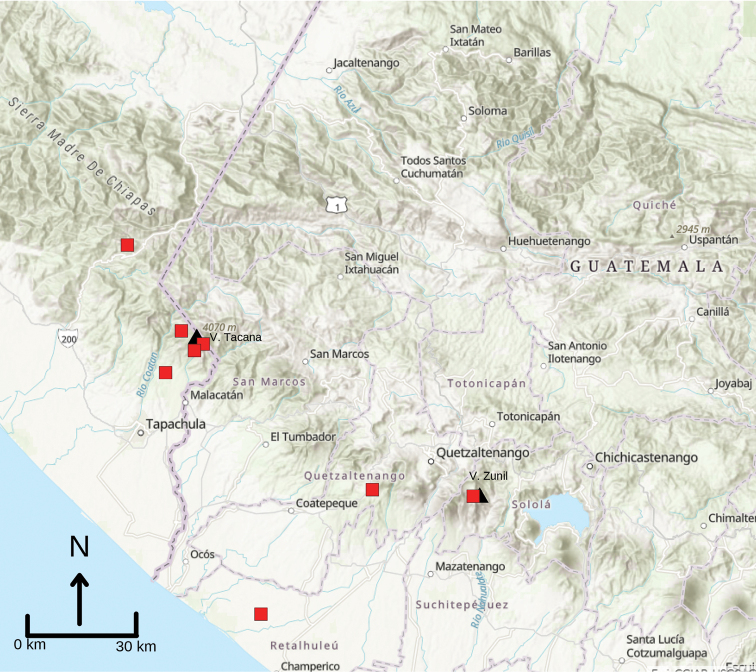
Map of the southern border regions of Guatemala and Chiapas, Mexico. Black triangles represent Volcán Tacaná and Volcán Zunil. Red squares represent museum specimens examined in this study. Note that some squares represent more than a single specimen. The record on the coastal plain of Quetzaltenango, Guatemala, is UMMZ 102472 which bears the locality Granja Lorena; we suspect that this is generalized locality information and the toad likely was collected to the north at higher elevation.

*Inciliustacanensis* has a small geographic distribution apparently restricted to moderately high elevations (ca 1500–1700 m) between the Cerro Mozotal, Chiapas, Mexico to the west to Volcán Zunil in Quetzaltenango, Guatemala to the east (Fig. 4). Despite considerable collecting efforts over many decades in Departamento San Marcos, Guatemala (reviewed by Rovito et al. 2009), no records are available from this intervening region. This distribution represents but a small portion of the Fuegan Faunal Area defined by Campbell and Vannini (1989a), and evidently does not include the Sierra Madre de Chiapas, Montañas de Cuilco, nor the Central Plateau of Chiapas. However, details of the geographic distribution of this species must be considered conservatively, as it is evident that this small, cryptic species is not readily encountered even in areas where it is known to occur. Detailed habitat notes are not available for any of the museum specimens, but the species apparently occurs in leaf litter in rainforest and cloudforest habitats.

In fact, information is lacking on various aspects of its biology, including intra and interspecific ecological interactions. It is known that the collection of the holotype (March 1938) and two paratypes (January 1940 and April 1949) was carried out in the dry season, although five of the paratypes were collected in the rainy season (August 1924 and 1950; Smith 1952).

This region is heavily cultivated in coffee, but we have no evidence that the species occurs in any form of coffee fields, unlike some other anurans in the area that can become quite abundant in areas of coffee production [e.g., *Craugastorrhodopis* (Cope, 1867) (Seib 1985)].

Oviductal eggs were present in females collected in July and August, suggesting that breeding occurs in the wet season. Clutch sizes were estimated (i.e., eggs were not removed and individually counted) between approximately 50–400 eggs. The eggs are small and pigmented.

*Inciliustacanensis* is superficially similar in size and sexual dimorphism to *I.epioticus* (Cope, 1875), *I.chompipe* (Vaughan and Mendelson 2007), and *I.guanacaste* (Vaughan & Mendelson, 2007) in Costa Rica. Those species have large (ca 5 mm diameter), unpigmented eggs and are now known to undergo direct development (Gray and Bland 2016). Unlike these diminutive (females about 35 mm SVL, males about 25 mm; Vaughan and Mendelson 2007) Costa Rican species, we presume that *I.tacanensis* has typical aquatic larvae. However, these larvae and all other aspects of reproduction in this species remain unknown.

The results of the real-time PCR analyses for the Bd pathogen for 15 specimens for 15 specimens of *I.tacanensis* collected between 1924–1978 and four *I.bocourti* from 1989–2012 all were negative (Table 2).

**Table 2.** Results of Real-Time PCR assays for amphibian chytridiomycosis (*Batrachochytriumdendrobatidis*; Bd) from preserved museum specimens of *Inciliusbocourti* and *I.tacanensis*. Complete locality data are listed in the Appendix 1.

Species	Specimen	Country	State	Date	Bd +/–
*I.bocourti*	MVZ 256842	Mexico	Chiapas	18 June 2012	–
*I.bocourti*	MVZ 256843	Mexico	Chiapas	25 June 2012	–
*I.bocourti*	UTA A-50918	Guatemala	Huehuetenango	17 June 1996	–
*I.bocourti*	UTA A-28855	Guatemala	Huehuetenango	29 July 1989	–
*I.tacanensis*	CAS 70691	Guatemala	Suchitepequez	3 August 1924	–
*I.tacanensis*	CAS 139889	Mexico	Chiapas	16 August 1974	–
*I.tacanensis*	FMNH 35063	Guatemala	Quetzaltenango	31 January 1940	–
*I.tacanensis*	UIMNH 24873	Mexico	Chiapas	- August 1950	–
*I.tacanensis*	UIMNH 55152	Mexico	Chiapas	30 July 1963	–
*I.tacanensis*	UIMNH 55153	Mexico	Chiapas	8 August 1963	–
*I.tacanensis*	UIMNH 55154	Mexico	Chiapas	8 August 1963	–
*I.tacanensis*	UIMNH 55155	Mexico	Chiapas	8 August 1963	–
*I.tacanensis*	UIMNH 55156	Mexico	Chiapas	8 August 1963	–
*I.tacanensis*	UIMNH 55157	Mexico	Chiapas	8 August 1963	–
*I.tacanensis*	UIMNH 55158	Mexico	Chiapas	8 August 1963	–
*I.tacanensis*	UIMNH 24874	Mexico	Chiapas	8 August 1963	–
*I.tacanensis*	UMMZ 102472	Guatemala	Quetzaltenango	21 April 1949	–
*I.tacanensis*	MVZ 170329	Mexico	Chiapas	30 July 1978	–
*I.tacanensis*	MVZ 170330	Mexico	Chiapas	30 July 1978	–

## ﻿Discussion

It appears that the last specimens (MVZ 191568–69) collected were found on Cerro Mozotal, Chiapas, Mexico on 22 October 1984 by Robert L. Seib. We know of no confirmed sightings or specimens since that time, despite considerable field work in the region over the subsequent decades by various teams. We know of no photograph of the species in life; one photograph of a living specimen (MVZ 264134) has been widely circulated on the internet, but in fact represents a mis-identified individual of *I.bocourti*.

*Inciliustacanensis* currently is listed as Endangered (criteria B1ab[iii]) on the Red List of Threatened Species of the International Union for Conservation of Nature (IUCN CSS Amphibian Specialist Group, 2020), but based on the guidelines for using the IUCN Red List categories and criteria (IUCN Standards and Petitions Committee, 2022) we suggest that the Red List be updated to include it in the Critically Endangered category, as the species is considered to be facing an extremely high risk of extinction in the wild, with populations of restricted distribution that are also severely fragmented by the continuous loss of habitat both in extent and quality, to the low number of historical localities from which the extant specimens were recorded, which is reflected in the absence of records in almost 40 years, and the presumed negative effects of amphibian chytridiomycosis caused by *Batrachochytriumdendrobatidis* (Bd). We also suggest that the Mexican federal government include the species in the Norma Oficial Mexicana (SEMARNAT 2010) in the risk category of endangered (P), based on criteria A, B, C and D of El Método de Evaluación del Riesgo de Extinción de las Especies Silvestres en México (MER), mainly considering the following aspects: for presenting a very restricted distribution (4 points) with little distribution in Mexico, less than 5% of the national territory; for occupying a hostile or very limiting habitat (3 points) with respect to the requirements for the natural development of the taxon; medium vulnerability (2 points), presenting a reproductive strategy where eggs and tadpoles are found in large to small bodies of lentic or lotic water; and the high human impact (4 points) due to the strong fragmentation of the habitat and the change in land use that occurs in the region. For the assignment of the risk category of endangered (P), the total ranges between 12 and 14 points.

With regards to the EVS, Wilson et al. (2013) and Johnson et al. (2015a, b) included *I.tacanensis* in the low category by assigning it a total of 9 points (4 for geographical distribution + 4 for ecological distribution + 1 point for the type of reproductive mode). The 4 points for ecological distribution consider that the species occurs in five vegetation formations; however, Johnson (1989) in his biogeographical analysis of the herpetofauna of the northwestern nuclear Central America mentions that the species is distributed in only two vegetation formations (lower montane rain forest and montane rain forest) for which it reaches a value of 7 points in the ecological distribution section, and a total of 12 considering the other aspects, for which it would be included in the medium category of the EVS that considers a range 10–13, even if three vegetation formations are considered by including the premontane tropical forest as different from the two formations already mentioned. It should be noted that Smith (1952) in the paper describing the species does not refer to the type of habitat or vegetation formation where the specimens were recorded, and there is no formally published information that considers various ecological aspects. Despite the proposal to change the category from low to medium, due to the argument that the species occurs in a smaller number of vegetation formations, it is necessary to point out the limitations of the EVS, in cases such as *I.tacanensis*, a taxon that has a limited distribution and is possibly extinct but is considered in the low category of this measure.

With regards to chytridiomycosis, we note that the timing of the last records, in 1984, corresponds closely to estimated epidemics in the region. Mendelson et al. (2014) estimated an outbreak of chytridiomycosis in the Sierra de las Minas, Guatemala, in 1983. Other reports of chytrid-induced declines from southern Mexico and Guatemala, similarly all are concentrated in the late 1970s and early 1980s (Lips et al. 2004; Rovito et al. 2009; Cheng et al. 2011; Scheele et al. 2019). It is perhaps noteworthy that extensive local collections on the slopes of Volcán Santa María, Quetzaltenango, Guatemala, in 1987 and 1988 (Campbell and Vannini 1989b) failed to discover this species. Although there are no historical records of *I.tacanensis* from this particular volcano, it is well within the estimated range of the species and bears seemingly appropriate habitats. Basanta et al. (2021) produced historical data for presence and distribution of Bd in Mexico. Their results indicate that Bd has been present in Mexico, in some genetic form, since at least the late 1800s, but their data indicate a drastic increase in prevalence during the period of 1970–1985, and further increasing afterwards.

The effects of chytridiomycosis on individuals and populations of *I.tacanensis* are completely unknown, but it is worth noting that some – but certainly not all – species in the genus are severely negatively affected (e.g., *I.periglenes*; Crump et al, 1992; Schachat et al., 2015). Muñoz Alonso (no date, probably 2010) reported that El Tacaná (15°02'10"N, 72°08'29"W, municipality of Cacahoatán), is one of 10 localities in Chiapas where chytridiomycosis has been recorded, confirmed in tree frogs *Plectrohylamatudai* Hartweg, 1941 and *P.sagorum* Hartweg, 1941; these localities occur at elevations ranging between 900 and 1200 m. These areas represent montane cloudforest habitats (bosque de pino) and semi-evergreen tropical forest (selva mediana subperennifolia). While our small and chronologically random sampling for Bd is inconclusive, considered together, one can envision a parsimonious scenario in which *I.tacanensis* was driven to extinction by Bd in the mid-1980s.

## Supplementary Material

XML Treatment for Incilius tacanensis

## ﻿Appendix 1

**Specimens of *I.tacanensis* verified by photographs or physical examination.**

The specimens here referred to *I.bocourti* and *I.luetkeni* had been catalogued in their respective collections as *I.tacanensis*.

*Inciliusbocourti*

Guatemala: Huehuetenango: Sierra de los Cuchumatanes, 30.4 km (by road) SSW San Juan Ixcoy (UTA A-28855); 5.1 km WSW Patacal (UTA A-50918). Mexico: Chiapas: Summit of Cerro Mozotal, Mpio. Motozintla (MVZ 272788); 1.8 km NE (by rd) of summit of Cerro Mozotal on road to Motozintla, Mpio. Motozintla (MVZ 256842); Ejido Boqueron, 14 km W (by road) of Niquivil, Mpio. Motozintla (MVZ 256843); Mpio. Motozintla, Ejido El Carrizal, Cerro el Mozotal (CZRHE 2603); Mpio. Motozintla, Pinabeto, alrededor del pueblo (CZRHE 2795); Mpio. Motozintla, cerca de Pinabeto, al sur del pueblo (CZRHE 2775); Mpio. El Porvenir, 0.79 km NE de Cañada, 5.32 km NW de El Porvenir (CZRHE 3010); Mpio. Motozintla, Ejido El Carrizal, Cerro El Mozotal (CZRHE 2598); Mpio. Motozintla, Ejido Libertad Calera (CZRHE 2823); Mpio. El Porvenir, El Porvenir (CZRHE 3014); Top of Cerro Tzontehuitz, near San Cristobal de las Casas (MVZ 264134).

*Inciliusluetkenii*

Guatemala: El Progreso: Morazan (AMNH 183098).

*Inciliustacanensis*

Guatemala: Suchitepequez: Volcán Zunil (CAS 70691); Quetzaltenango: Finca Montecristo, Rio Samala (FMNH 35063); Granja Lorena (UMMZ 102472). Mexico: Chiapas: 1500 m on Volcán de Tacaná (UMMZ 88359); 8 km N Juárez (KU 94009); Colonia Talquian, Volcán Tacaná (MVZ 159445–48); Volcán Tacaná, above Cacahuatan (UIMNH 6177–78, 24873–74); Union Juárez (UIMNH 55152); near Talquian (UIMNH 55152–58); Volcán Tacaná, 3 km N of Union Juárez (CAS 139889–90); Colonia Talquian, 3 km N (by road) Union Juárez, Volcán Tacaná (MVZ 170329, 170330); Cerro Mozotal, 16.7 mi (via road to Siltepec) from pass on continental divide above Huixtla (MVZ 191569); Volcán de Tacaná, above Cacahuatan (USNM 139721); Union Juárez, Ejido Talquian y Chiquihuites (IBUNAM-CNAR 5407, 2 specimens).
